# Radial Compressive Property and the Proof-of-Concept Study for Realizing Self-expansion of 3D Printing Polylactic Acid Vascular Stents with Negative Poisson’s Ratio Structure

**DOI:** 10.3390/ma11081357

**Published:** 2018-08-06

**Authors:** Zichao Wu, Ji Zhao, Wenzheng Wu, Peipei Wang, Bofan Wang, Guiwei Li, Shuo Zhang

**Affiliations:** School of Mechanical and Aerospace Engineering, Jilin University, Changchun 130025, China; wuzc15@mails.jlu.edu.cn (Z.W.); jzhao@jlu.edu.cn (J.Z.); wangpe1_i@163.com (P.W.); wangbf1415@mails.jlu.edu.cn (B.W.); ligw15@mails.jlu.edu.cn (G.L.); zhangshuo028@jlu.edu.cn (S.Z.)

**Keywords:** 3D printing, polylactic acid, vascular stent, negative Poisson’s ratio, shape memory effect

## Abstract

Biodegradable stents offer the potential to reduce the in-stent restenosis by providing support long enough for the vessel to heal. The polylactic acid (PLA) vascular stents with negative Poisson’s ratio (NPR) structure were manufactured by fused deposition modeling (FDM) 3D printing in this study. The effects of stent diameter, wall thickness and geometric parameters of arrowhead NPR structure on radial compressive property of 3D-printed PLA vascular stent were studied. The results showed that the decrease of stent diameter, the increase of wall thickness and the increase of the surface coverage could enhance the radial force (per unit length) of PLA stent. The radial and longitudinal size of PLA stent with NPR structure decreased simultaneously when the stent was crimped under deformation temperature. The PLA stent could expand in both radial and longitudinal direction under recovery temperature. When the deformation temperature and recovery temperature were both 65 °C, the diameter recovery ratio of stent was more than 95% and the maximum could reach 98%. The length recovery ratio was above 97%. This indicated the feasibility of utilizing the shape memory effect (SME) of PLA to realize the expansion of 3D-printed PLA vascular stent under temperature excitation.

## 1. Introduction

Vascular stent implantation is one of the main therapies used to treat vascular occlusion diseases [1]. In-stent restenosis is the re-blockage of vascular stent after stent implantation, which is the main failure form of stents and a key problem remaining to be solved [2]. Bare metal stents easily cause damage to the blood vessel wall and non-degradable metal stents may lead to rejection reactions [3]. Drug-eluting stents depend on antiproliferative drugs to prevent repairing of damaged vascular endothelium, which delays the in-stent restenosis but cannot fundamentally solve the problem of stent restenosis [4,5]. Biodegradable stents can generally degrade in the human body after expansion and support of narrow vessels. Biodegradable stents can eliminate the problem of long-term rejection of the stents and avoid the damage and stimulation of the surrounding vessel wall, which effectively solves the problem of in-stent restenosis [6,7,8].

Polylactic acid (PLA) is a non-irritant and high-strength thermoplastic aliphatic polyester possessing superior biocompatibility and biodegradability [9]. PLA is also a thermal-induced physically cross-linked shape memory polymer [10]. The shape memory effect (SME) of PLA results from a combination of polymer structure and morphology, which consists of two segregated sets of domains: the crystalline domains as the fixed phase, and the amorphous domains as the reversible phase [11]. A full cycle of the shape-memory procedure consists of three stages of material shape: original shape, temporary shape and original shape recovery. Because of the good mechanical strength, biocompatibility and the SME, PLA has good application prospects in many biomedical fields such as vascular stent, drug release, thrombus removal, surgical suture and tissue engineering scaffolds [12,13,14]. Stack et al. [15] implanted biodegradable Poly-l-Lactic Acid (PLLA) scaffolds into experimental dogs. Preliminary results showed that the stent only partly caused a small amount of thrombus and intimal hyperplasia in the short term. Agrawal et al. [16] evaluated the PLLA monofilaments for use as intravascular polymeric stents. Stents constructed with similar monofilaments were tested under hydrostatic pressure, and the results correlated with the properties of the monofilaments. Stent collapse pressure was a decreasing function of stent diameter and filament draw ratio. Tamai et al. [17] first applied PLLA stent with a thickness of 0.17 mm a zigzag helical coil pattern to human body. A total of 25 stents were successfully implanted in 19 lesions in 15 patients. No stent thrombosis and no major cardiac event occurred within 30 days. Their preliminary experience suggested that coronary PLLA biodegradable stents were feasible, safe, and effective in humans. Venkatraman et al. [18] produced helical PLLA stents with different molecular weights. The effects of molecular weight, drug incorporation and stent design on the collapse pressure of the stents were evaluated. The results showed that the range of molecular weight of the polymers used did not significantly affect the collapse pressure. Drug incorporation, in high doses, reduced the mechanical properties. And the mechanical integrity of the stents did not change over 6 weeks.

Three-dimensional printing, also known as additive manufacturing, is a method of manufacturing 3D solid parts by bottom-up layer-by-layer accumulation on the basis of computer-aided design and computer-aided manufacturing [19,20]. It has obvious advantages of cost and efficiency in the manufacturing of small-batch, customized and individualized complex products, which is suitable for manufacturing of vascular stent [21]. 3D printing can also overcome the disadvantages of traditional processing methods such as low degree of automation, high cost, requirement of many post-processing technologies, difficulty in processing complex structure and so on [22,23]. Kaesemeyer et al. [24] used a rapid stent fabrication (RSF) with extrusion system and a four-axis motion system to fabricated bioresorbable polystatin stents comprising lactide, glycolide, caprolactone, and lovastatin (60:15:10:15 parts by weight). It preliminarily appeared feasible to fabricate bioresorbable stents that had the potential to deliver two drugs to the site of the procedure-related vessel lumen injury. Park et al. [25] prepared a helical, biocompatible, and biodegradable stent by 3D rapid prototyping and implanted stent into the porcine femoral artery. The fabricated polycaprolactone (PCL) stent was coated with sirolimus mixed with poly-(lactide-co-glycolide) (PLGA) and polyethylene glycol (PEG), via a spraying method for slow drug release. It was found to reduce neointimal hyperplasia and lead to a desirable low fibrin score, which suggested that 3D-printing could be utilized for the fabrication of drug-eluting stents. Robert van Lith et al. [26] synthesized a photocurable, antioxidant and bioresorbable citrate based biomaterial and fabricated stents by a custom-made micro-continuous liquid interface production system (microCLIP). The stent strength and dimensional accuracy were characterized. The rapid and high-resolution bioabsorbable stent was customized.

PLA vascular stents with arrowhead NPR structure were fabricated by FDM 3D printing technology in this study. The effects of stent diameter, wall thickness and geometric parameters of arrowhead NPR structure on radial compressive property of 3D-printed PLA vascular stents were studied. Using a self-made crimping device and constant-temperature numerical control water-bath box, we studied the SME and NPR effect of the PLA stent. The dimensional changes of the length and diameter were measured. The length recovery ratio and diameter recovery ratio were calculated.

## 2. Experiment and Methods

### 2.1. Establishment of Vascular Stent Model with NPR Structure

Most vascular stent models researched in current related studies had relatively simple structures such as Z-shaped structure, spiral structure and corrugated structure. The exploration of the new structure of vascular stent still needs further research. The concept of Poisson’s ratio is discovered and proposed by the French scientist Poisson, which is defined as the ratio of the lateral strain to the longitudinal strain under the single axis tension or compression. The Poisson’s ratio can be expressed as the following Formula (1):

*γ* = −*ε_i_*/*ε_j_* (1)

*γ* represents Poisson’s ratio, *ε_i_* represents lateral strain, *ε_j_* represents longitudinal strain, *i* and *j* represent two perpendicular axes respectively [27]. The NPR structure has a unique auxetic behavior, that is, when subjected to uniaxial tension or compression, both lateral strain and longitudinal strain are positive or negative. Many NPR structures have been proposed successively with the study of the NPR structure, such as the re-entrant hexagonal structure, the star honeycomb structure, the arrowhead structure, the chiral honeycomb structure and the rotating quadrangle structure [28]. Materials and structures with NPR effect have good application in biomedicine, aerospace, shock absorption, sound insulation, energy absorption and cushioning fields [29]. Applying NPR structure to vascular stent can reduce the radial and longitudinal size of stent simultaneously when the stent is crimped, which is beneficial to minimally invasive implantation. And the vascular stent can expand in both radial and longitudinal direction, which allows good anchorage with arterial walls [30].

**Figure 1:**
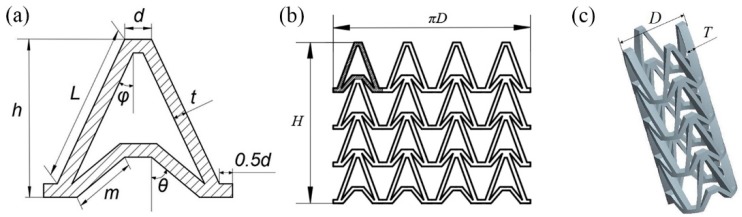
Vascular stent model with NPR structure, (**a**) Arrowhead NPR structure unit; (**b**) Stent flat developing drawing; (**c**) Three-dimensional model of stent.

In this study, an NPR structure was used as the structural unit of the stent, which made the stent have NPR effect in radial and longitudinal direction. The Autodesk Computer Aided Design (AutoCAD) was used to draw arrowhead structure unit as shown in Figure 1a. The arrowhead structure unit was repeated along the axis and circumferential directions to form the three-dimensional model of the vascular stent. The flat developing drawing of the stent is shown in Figure 1b and the three-dimensional model of the stent is shown in Figure 1c.

The geometry of the vascular stent can be defined by the size parameters shown in Figure 1:

- *L* represents the length of the support strut;
- *m* represents the length of the re-entrant strut;
- *h* represents the height of the unit;
- *t* represents the width of the strut;
- *d* represents the width of the link;
- *φ* represents the angle between the support strut and the axial direction;
- *θ* represents the angle between the re-entrant strut and the axial direction;
- *D* represents the outer diameter of the vascular stent;
- *H* represents the total length of vascular stent;
- *T* represents the thickness of the stent.

### 2.2. Experimental Facility and Materials

The PLA vascular stents with NPR structure were prepared by Ultimaker 3 Extended FDM 3D printer (Ultimaker B.V., Geldermalsen, Netherlands). The Ultimaker 3 Extended 3D printer has a dual extrusion system, which can print two different materials. The nozzle 1 prints the natural PLA material, and the nozzle 2 prints the water-soluble support material Polyvinyl Alcohol (PVA). After printing, soaking the three-dimensional solid in water will remove the support materials and leave the required three-dimensional solid products. The 3D printing filaments of PLA and PVA were purchased from Esun (Shenzhen Esun Industrial Co., Ltd., Shenzhen, China). The natural PLA is colorless and slightly transparent and has the density of 1.24 g/cm^3^, the melting index of 5 g/10 min (190 °C/2.16 kg), the tensile strength of 65 MPa, the elongation at break of 8%, the bending strength of 97 MPa, the flexural modulus of 3.6 GPa, the impact strength of 4 kJ/m^2^. PVA is soluble in water and has the density of 1.25 g/cm^3^, the tensile strength of 22 MPa, the elongation at break of 360%.

### 2.3. 3D Printing of PLA Vascular Stents

**Figure 2:**
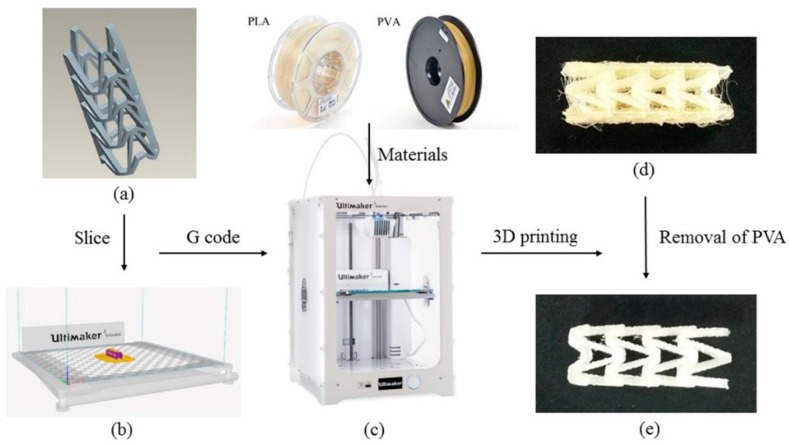
3D printing process of PLA vascular stent, (**a**) The three-dimensional model of vascular stent; (**b**) The Cura software interface; (**c**) The PLA, PVA filaments and Ultimaker 3 Extended 3D printer; (**d**) The PLA vascular stent with PVA support material; (**e**) The PLA vascular stent removed PVA material.

The 3D printing process of PLA vascular stent is shown in Figure 2. The three-dimensional model of vascular stent was sliced by Cura slice software (V2.6.1, Ultimaker B.V., Geldermalsen, The Netherlands) as shown in Figure 2b. The nozzle 1 printed the natural PLA material, and the nozzle 2 printed the water-soluble support material PVA. The 3D printing parameters were identical for each PLA vascular stent. The nozzle diameter was 0.4 mm, the layer thickness was 150 μm, the raster angle was 45°, the number of contours was 1 contour, the infill density was 100%, and the build plate temperature was 60 °C. The printing temperature of PLA was 200 °C and the printing speed was 65 mm/s. The printing temperature of PVA was 220 °C and the printing speed was 35 mm/s. In order to reduce the surface staircase defects caused by the excessive number of layers and cohere the PLA vascular stent to the build plate, the printing direction of the stent was horizontal. The PLA vascular stent was placed in center of the build plate to eliminate the temperature difference caused by the position factors. The 3D printing filaments of PLA, PVA and the Ultimaker 3 Extended 3D printer are shown in Figure 2c. The PLA vascular stent with PVA support material is shown in Figure 2d. After immersed in water until the water-soluble PVA was dissolved completely, the PLA vascular stent was obtained as shown in Figure 2e.

**Figure 3:**
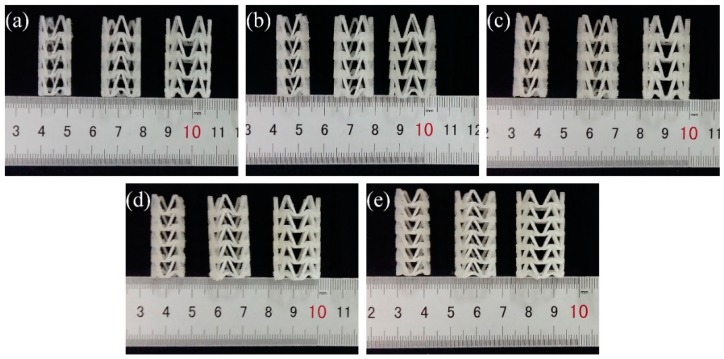
3D-printed PLA vascular stents, from (**a**–**e**) are 3D-printed PLA vascular stents of A, B, C, D and E group respectively.

In order to study the influence of stent diameter, wall thickness and geometric parameters of the arrowhead NPR structure on the radial compressive property of 3D-printed PLA vascular stent, five groups of vascular stents of A, B, C, D and E were printed respectively. The experimental groups and the size parameters of the stents are shown in Table 1. And the 3D-printed PLA vascular stents with NPR structure of A, B, C, D and E group are shown in Figure 3a–e respectively.

### 2.4. Compression Experimental Process of PLA Vascular Stents

**Figure 4:**
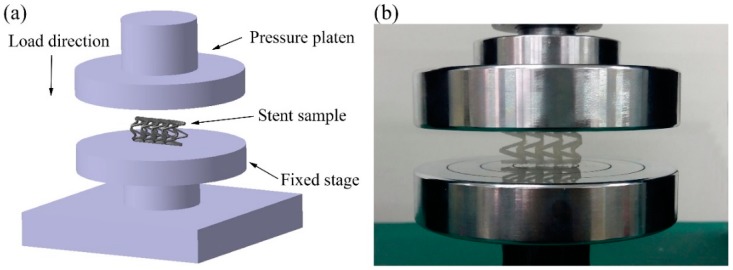
Compression experiment of PLA vascular stents, (**a**) the diagram of loading test setup; (**b**) stent sample in the test.

The compression experiment was tested by a UTM6104 microcomputer-controlled electronic universal testing machine (Shenzhen Suns Technology Stock Co., Ltd., Shenzhen, China). As shown in Figure 4, the PLA vascular stent was placed horizontally on the stage and the pressure platen was compressed at the speed of 1 mm/min until the stent was crushed. The compressive force was applied following the radial direction of the stent, which was perpendicular to the axis of the stent. The radial force versus platen displacement was measured during the compression process. In order to facilitate the comparison of different lengths and geometric parameters of stents, the radial force was divided by the total length of the stent for normalization. The radial force of the stent (per unit length) F (N/mm) was measured as a function of radial displacement Dis (mm) [31].

### 2.5. SME Experimental Process of PLA Vascular Stents

The proof-of-concept study for self-expansion by SME of 3D-printed PLA vascular stents with NPR structure was carried out by self-made crimping device and constant-temperature numerical control water-bath box. The change of diameter and length of stent after deformation and recovery were measured, the diameter recovery ratio and length recovery ratio were obtained.

**Figure 5:**
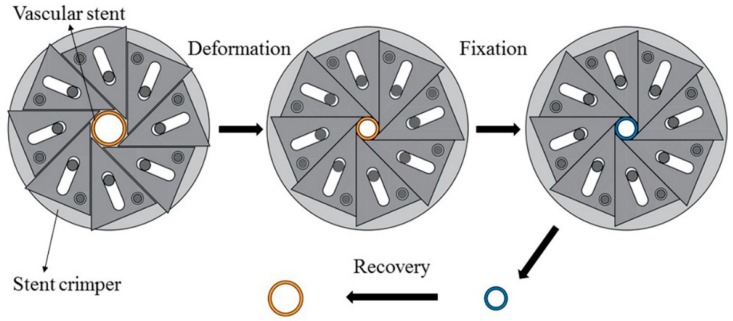
SME experimental process of PLA vascular stent.

Before the experiment, the initial diameter *D_O_* and the initial length *H_O_* of PLA stent were measured. The experimental process mainly included three parts as shown in Figure 5. The first part was the deformation of the PLA stent, in which a crimped shape was created. Water in the constant-temperature numerical control water-bath box was heated to the deformation temperature of 65 °C. The PLA stent was placed in a water-bath box for 5 min and was then crimped by using a self-made crimping device. The diameter of PLA stent was decreased gradually and the crimping process was stopped until the circumferential structure units were deformed and close to each other. The second part was the fixation of the deformed PLA stent. The PLA stent and crimping device was removed from the water-bath box maintained the crimped shape and cooled down to room temperature. The PLA stent was taken out of the crimping device after completely cooled and fixed. The crimped diameter *D_C_* and crimped length *H_C_* of PLA stent were measured. The third part was the recovery of the PLA stent, in which the original shape of the PLA stent was restored. The water temperature in the constant-temperature numerical control water-bath box was set to the recovery temperature of 65 °C, and the crimped PLA stent was then placed in the water-bath box until the shape didn’t change. The recovery diameter *D_r_* and the recovery length *H_r_* of PLA stent were measured. According to Formulas (2) and (3), the diameter recovery ratio and length recovery ratio of 3D-printed PLA stent were calculated.

*R_D_* = *D_r_*/*D_O_* (2)

*R_H_* = *H_r_*/*H_O_* (3)

*R_D_* and *R_H_* represent the diameter recovery ratio and length recovery ratio of 3D-printed PLA stent respectively.

**Figure 6:**
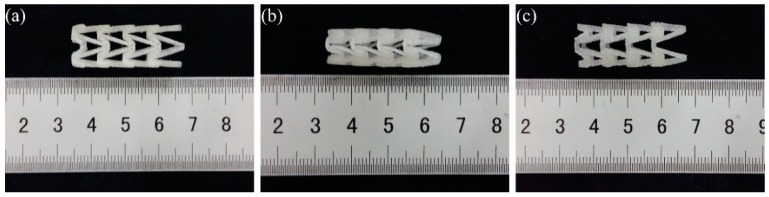
Shape change of numbered B1 3D-printed PLA vascular stent with NPR structure, (**a**) Original PLA stent; (**b**) Crimped PLA stent; (**c**) Recovery PLA stent.

The shape change of numbered B1 3D-printed PLA vascular stent in SME experiment is shown in Figure 6. Figure 6a shows the original shape of the PLA stent before experiment and b shows the PLA stent after deformed under deformation temperature and fixed under room temperature. Figure 6c shows the PLA stent after recovery under recovery temperature.

## 3. Results and Discussion

### 3.1. Compression Experimental Results

**Figure 7:**
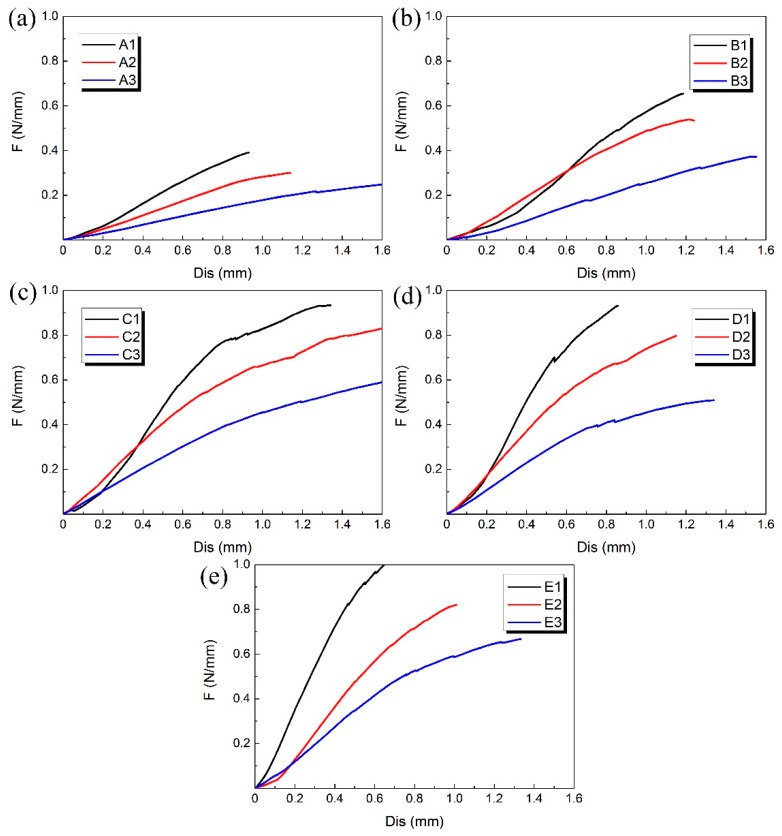
Radial force (per unit length) versus radial displacement curves for different groups of 3D-printed PLA vascular stents: from (**a**–**e**) are A, B, C, D and E group respectively.

The compression experiment was performed on each 3D-printed PLA vascular stent, and the results of each group are shown in Figure 7. The 3 PLA stents in each group had the same wall thickness and geometric parameters of arrowhead NPR structure, but had different diameters.

At the beginning of the curves, the slopes were relatively large and the increase of the radial deformation of the stents was relatively slow. When the radial displacement was less than 0.3 mm, the difference of compressive property between the 3D-printed PLA stents with different diameters in each group is relatively small. However, as the compressive force increased, the curves gradually became relatively smooth and the effect of different diameters on radial force of stents became more significant. At the stage of small radial displacement, the deformation of vascular stent was mainly elastic deformation. As the deformation increased, the plastic deformation appeared and the stent was in the stage of plastic instability. The radial deformation of the stent was more obvious.

The A1, B1, C1, D1 and E1 stent with the minimum diameter of 12 mm in each group of PLA stent had the highest radial force and the best ability to resist compressive deformation. With the increase of the stent diameter, the radial force was reduced and the ability to resist compressive deformation became worse. That is, the 3D printed PLA vascular stent with same wall thickness and geometric parameters of arrowhead NPR structure had the higher radial force and better ability to resist compressive deformation as the stent diameter decreased. Because the main deformation occurred on the two sides of the vascular stent, the arm of force between easily-deformed side and middle part subjected to compressive load became longer with the increase of the stent diameter. The force needed for the plastic deformation of the stent was smaller, which made the stent more vulnerable to instability and the ability to resist compressive deformation decreased correspondingly.

**Figure 8:**
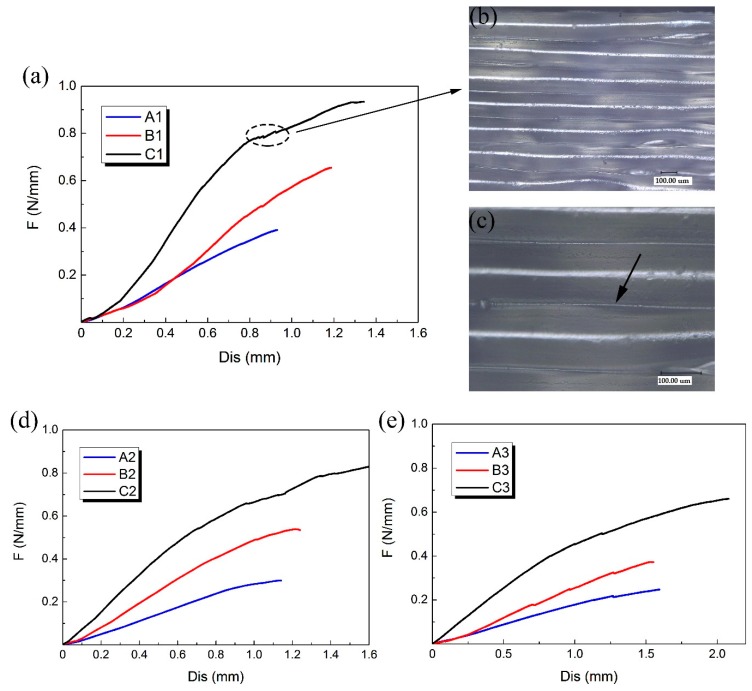
Radial force (per unit length) versus radial displacement curves for different wall thickness of 3D-printed PLA vascular stents and the digital microscope images of stent surface, (**a**) PLA vascular stents with 12 mm stent diameter; (**b**) Digital microscope image of PLA stent surface magnified 200 times; (**c**) Digital microscope image of PLA stent surface magnified 500 times; (**d**) PLA vascular stents with 15 mm stent diameter; (**e**) PLA vascular stents with 18 mm stent diameter.

A, B and C three groups of 3D-printed PLA vascular stents had the same geometric parameters of arrowhead NPR structure, but different wall thicknesses. Compared the PLA stents with the same stent diameter in the three groups of A, B and C to study the effect of different wall thickness on the radial compressive property of PLA stent. The radial force versus radial displacement curves for different wall thickness are shown in Figure 8a,d,e. In the case of small deformation, the effect of the wall thickness on the radial force of the stent was not significant. Under the same compressive force, the difference of deformation between stents with different wall thicknesses was relatively small. However, with the increase of compressive force, the wall thickness showed a prominent role in compressive property. The difference of deformation between stents with different wall thicknesses became more and more notable.

The 3D-printed PLA vascular stents of C group had the wall thickness of 1.8 mm, which had the strongest ability to resist radial deformation and the highest radial force compared with the A group of 1.2 mm wall thickness and B group of 1.5 mm wall thickness. The increase of the wall thickness made the cross sectional area of the support strut of the stent increase. As the wall thickness increased, the ability to resist radial deformation and the radial force of 3D-printed PLA vascular stents with same geometric parameters of arrowhead NPR structure and stent diameter became stronger and higher.

A few discontinuities were found in the response curves as shown in the dotted line frame in Figure 8a. These discontinuities occurred when some supporting struts were locally fractured during compression test. The digital microscope image of surface of the PLA stent with 1.8 mm wall thickness magnified 200 times and 500 times are shown in Figure 8b,c, respectively. The surface of the PLA stent manufactured by FDM was stratified. Each layer presented a semicircular convex shape, while the two adjacent layers had a concave shape, forming a small groove between two adjacent layers as the part pointed by the arrow in Figure 8c. This is because that the forming method of FDM is to melt the printing filament and then accumulate the melted material layer-by-layer to form 3D solid parts. The combination between layer and layer is realized by bonding solid material and melted material. The staircase effect caused by the layered manufacturing [32] is easy to cause small groove between the two adjacent layers, which results in stress concentration. The tiny cracks caused by stress concentration were easily emerged from small grooves during the compressive process, which led to the break of the stent support struts and decrease of the mechanical property. The stress concentration caused by the staircase shape between the layers had more significant impact on the 3D-printed PLA stents with smaller wall thickness and was one of the reasons for the easier fracture of the stent with smaller wall thickness during the compression test.

**Figure 9:**
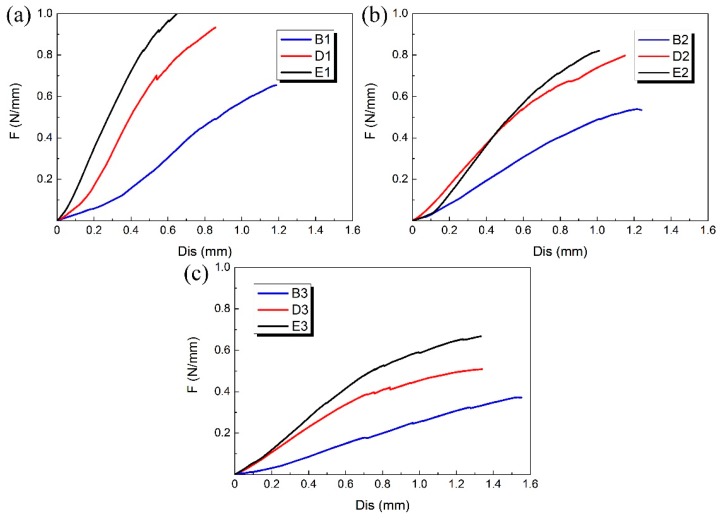
Radial force (per unit length) versus radial displacement curves for different geometric parameters of arrowhead NPR structure of 3D-printed PLA vascular stents: (**a**) PLA vascular stents with 12 mm stent diameter; (**b**) PLA vascular stents with 15 mm stent diameter; (**c**) PLA vascular stents with 18 mm stent diameter.

The B, D and E groups of 3D-printed PLA vascular stents had the same wall thickness, but different geometric parameters of arrowhead NPR structure. We compared the PLA stents with the same stent diameter in the three groups of B, D and E to study the effect of geometric parameters of arrowhead NPR structure on the radial compressive property of PLA stent. The radial force versus radial displacement curves for different wall thickness are shown in Figure 9a–c. The PLA stents in group E had the highest radial force, followed by D group and B group had the smallest. In condition of same stent diameter, the increase of angle *φ* and *θ* made the length *L*, *m* and *h* shorter, which led to the increase of surface coverage of the stent. Under the same compressive load, the stent with larger surface coverage had stronger compressive property and more material was deformed to resist deformation.

### 3.2. SME Experimental Results

The initial diameter *D_O_* and length *H_O_*, the crimped diameter *D_C_* and length *H_C_*, the recovery diameter *D_r_* and length *H_r_* were measured and the diameter recovery ratio *R_D_* and the length recovery ratio *R_H_* were calculated. The SME experimental results are shown in Table 2.

The values of *D_C_* and *H_C_* were greater than those of *D_O_* and *H_O_*, which indicated that the diameter and length of 3D-printed PLA vascular stent with arrowhead NPR structure decreased at the same time when the stent was crimped under deformation temperature. Also the *D_r_* and *H_r_* were greater than *D_C_* and *H_C_*. The diameter and length of the stent increased simultaneously when the stent was expanded. The experimental results showed that by utilizing arrowhead NPR structure, the radial and longitudinal size of the PLA stent could reduce at the same time, which was beneficial to achieve minimally invasive implantation and deliver easily to the lesion part. And the increase of radial and longitudinal size of the PLA stent would avoid the foreshortening and allow good anchorage with arterial walls.

The results showed that 3D-printed PLA vascular stents had favorable SME. When the deformation and recovery temperature were both 65 °C, the *R_D_* of each stent was above 95% and up to 98%. The *R_H_* was above 97%, which basically restored to the original shape. However, different structural parameters have no obvious effect on SME of 3D-printed PLA vascular stent.

The experimental results showed that it was feasible to use the SME of PLA to realize self-expansion of vascular stent under temperature excitation. Although the selection of PLA shape memory recovery temperature was higher than the normal body temperature of 37 °C, with the further research on the shape memory polymers, the recovery temperature could be adjusted by material modification and other methods. The shape memory experiment of 3D-printed PLA vascular stents still showed that 3D-printed PLA stents had a very good application prospect in the biomedical field.

## 4. Conclusions

In this paper, the PLA vascular stents with arrowhead NPR structure were manufactured by Ultimaker 3 Extended FDM 3D printer. The effects of stent diameter, wall thickness and geometric parameters of arrowhead NPR structure on radial compressive property of 3D-printed PLA vascular stents were studied by compression experiments. With the increase of stent diameter, the per unit length of radial force of PLA stents decreased, and the stents with the minimum diameter of 12 mm in each group had the highest radial force and the best ability to resist compressive deformation. The wall thickness had the most significant effect on the mechanical property of PLA stents. The increase of wall thickness enhanced compressive property of PLA stent and the stents with 1.8 mm wall thickness had the highest radial force. The change of geometric parameters led to a change of the surface coverage of the stent, and the increase of the surface coverage made the radial compressive property better. In this proof-of-concept study, the SME of PLA stents was studied. The dimensional changes of the length and diameter of PLA stents were measured before and after the SME experiment. The diameter recovery ratio and the length recovery ratio were calculated. In the SME experiments, the PLA stent showed good NPR effect that the length and diameter of PLA stent could shrink and expand simultaneously. The NPR effect of PLA stent was beneficial to minimally invasive implantation and good anchorage with vessel walls. In the condition that both the deformation temperature and recovery temperature were 65 °C, the diameter recovery ratio was above 95%, the maximum was up to 98%, and the length recovery ratio was above 97%. Although the glass transition temperature of PLA is higher than human body temperature, the feasibility of using shape memory function of PLA to restore the original shape under the temperature excitation was verified. It was proved that the SME could be used to realize the self-expansion of the stent under temperature excitation.

## Figures and Tables

**Table 1:** Experimental groups and size parameters of PLA vascular stents.

Group	Number	*D*/mm	*H*/mm	*T*/mm	*L*/mm	*m*/mm	*h*/mm	*t*/mm	*d*/mm	*φ*/°	*θ*/°
A	A1	12	30.92	1.2	9.39	3.21	9.57	0.75	1.5	20	40
	A2	15	30.92	1.2	9.39	3.21	9.57	0.75	1.5	20	40
	A3	18	30.92	1.2	9.39	3.21	9.57	0.75	1.5	20	40
B	B1	12	30.92	1.5	9.39	3.21	9.57	0.75	1.5	20	40
	B2	15	30.92	1.5	9.39	3.21	9.57	0.75	1.5	20	40
	B3	18	30.92	1.5	9.39	3.21	9.57	0.75	1.5	20	40
C	C1	12	30.92	1.8	9.39	3.21	9.57	0.75	1.5	20	40
	C2	15	30.92	1.8	9.39	3.21	9.57	0.75	1.5	20	40
	C3	18	30.92	1.8	9.39	3.21	9.57	0.75	1.5	20	40
D	D1	12	31.10	1.5	7.60	2.76	7.64	0.75	1.5	25	50
	D2	15	31.10	1.5	7.60	2.76	7.64	0.75	1.5	25	50
	D3	18	31.10	1.5	7.60	2.76	7.64	0.75	1.5	25	50
E	E1	12	31.67	1.5	6.42	2.47	6.31	0.75	1.5	30	60
	E2	15	31.67	1.5	6.42	2.47	6.31	0.75	1.5	30	60
	E3	18	31.67	1.5	6.42	2.47	6.31	0.75	1.5	30	60

**Table 2:** SME experimental results of 3D-printed PLA vascular stents.

Group	Number	*D_O_*/mm	*H_O_*/mm	*D_C_*/mm	*H_C_*/mm	*D_r_*/mm	*H_r_*/mm	*R_D_*	*R_H_*
A	A1	12.20	31.04	8.60	30.11	11.75	30.42	0.9631	0.9800
	A2	15.13	31.03	10.38	29.76	14.76	30.39	0.9755	0.9794
	A3	17.92	31.12	12.39	29.67	17.41	30.25	0.9715	0.9720
B	B1	12.10	30.99	9.22	30.03	11.74	30.39	0.9702	0.9806
	B2	15.16	30.94	10.89	29.59	14.54	30.29	0.9591	0.9790
	B3	18.10	31.08	13.31	29.83	17.41	30.27	0.9619	0.9739
C	C1	12.22	30.92	9.91	29.97	11.64	30.53	0.9525	0.9874
	C2	15.19	31.04	10.84	29.55	14.62	30.46	0.9625	0.9813
	C3	18.12	30.96	12.21	28.83	17.84	30.39	0.9845	0.9816
D	D1	12.09	31.26	8.62	29.19	11.73	30.40	0.9702	0.9725
	D2	15.14	31.24	11.02	28.98	14.63	30.54	0.9663	0.9776
	D3	18.14	31.34	11.31	28.12	17.68	30.51	0.9746	0.9735
E	E1	12.13	31.82	9.21	30.66	11.71	31.07	0.9654	0.9764
	E2	15.26	31.71	10.79	29.50	14.57	30.80	0.9548	0.9713
	E3	18.14	31.76	13.03	29.42	17.72	30.83	0.9768	0.9707
